# Microbial community structure in an uranium-rich acid mine drainage site: implication for the biogeochemical release of uranium

**DOI:** 10.3389/fmicb.2024.1412599

**Published:** 2024-06-26

**Authors:** Xinxiang Wei, Hongliang Chen, Fangfang Zhu, Jiang Li

**Affiliations:** ^1^School of Water Resources and Environmental Engineering, East China University of Technology, Nanchang, Jiangxi, China; ^2^Department of Hydraulic Engineering, Jiangxi Water Resource Institute, Nanchang, Jiangxi, China; ^3^College of Nursing Health Sciences, Yunnan Open University, Kunming, Yunnan, China; ^4^State Key Laboratory of Nuclear Resources and Environment, East China University of Technology, Nanchang, Jiangxi, China; ^5^Teachers’ College, East China University of Technology, Nanchang, Jiangxi, China

**Keywords:** acid mine drainage, bacterial community, uranium, release, stone coal mine

## Abstract

The generation of acid mine drainage (AMD) characterized by high acidity and elevated levels of toxic metals primarily results from the oxidation and dissolution of sulfide minerals facilitated by microbial catalysis. Although there has been significant research on microbial diversity and community composition in AMD, as well as the relationship between microbes and heavy metals, there remains a gap in understanding the microbial community structure in uranium-enriched AMD sites. In this paper, water samples with varying levels of uranium pollution were collected from an abandoned stone coal mine in Jiangxi Province, China during summer and winter, respectively. Geochemical and high-throughput sequencing analyses were conducted to characterize spatiotemporal variations in bacterial diversity and community composition along pollution groups. The results indicated that uranium was predominantly concentrated in the AMD of new pits with strong acid production capacity, reaching a peak concentration of 9,370 μg/L. This was accompanied by elevated acidity and concentrations of iron and total phosphorus, which were identified as significant drivers shaping the composition of bacterial communities, rather than fluctuations in seasonal conditions. In an extremely polluted environment (pH < 3), bacterial diversity was lowest, with a predominant presence of acidophilic iron-oxidizing bacteria (such as *Ferrovum*), and a portion of acidophilic heterotrophic bacteria synergistically coexisting. As pollution levels decreased, the microbial community gradually evolved to cohabitation of various pH-neutral heterotrophic species, ultimately reverting back to background level. The pH was the dominant factor determining biogeochemical release of uranium in AMD. Acidophilic and uranium-tolerant bacteria, including *Ferrovum*, *Leptospirillum*, *Acidiphilium*, and *Metallibacterium,* were identified as playing key roles in this process through mechanisms such as enhancing acid production rate and facilitating organic matter biodegradation.

## 1 Introduction

Acid mine drainage (AMD) is the biggest environmental problem facing the global mining industry, characterized by strong acidity and high concentrations of sulfates and toxic metals, thereby posing a severe risk of environmental contamination in proximity to mining sites and presenting a persistent threat to ecosystems (Acharya and Kharel, 2020; Aguilar-Garrido et al., 2023). It is produced through the oxidation of sulfide minerals (e.g., pyrite), and the total reaction process is as follows: FeS_2_ + 3.5O_2_ + H_2_O → Fe^2+^ + 2SO_4_^2−^ + 2H^+^ (Baker and Banfield, 2003). Mining operations increase the surface area of sulfides exposed to air and water, thereby accelerating the rate of AMD generation (Galhardi and Bonotto, 2016). The sustained oxidation of sulfide minerals relies on the recycling and regeneration of Fe^3+^, but under acidic conditions (pH < 4), the rate of abiotic oxidation from Fe^2+^ to Fe^3+^ is very low, which limits the production of AMD (Baker and Banfield, 2003). However, some iron-oxidizing bacteria and archaea in AMD can catalyze this process (Edwards et al., 2000; Chen et al., 2016), accelerating the rate of Fe^2+^ oxidation by five orders of magnitude (Singer and Stumm, 1970). Thus, microbes play a crucial role in the production of AMD.

Changes in community structure are influenced by various environmental factors (e.g., the concentrations of toxic heavy metals or metalloids) because heavy metals can bind to microbial proteins, enzymes, and nucleic acids, interfering with their normal functioning and leading to toxicity (Fashola et al., 2016). A large number of AMD sites with microbial communities shaped by concentrations of heavy metals/metalloids have been reported, e.g., Hengshi River contaminated by Cu-Cd-Pb-Zn from the Dabao Shan Mine in Guangdong, China (Zhang et al., 2019), sulfide-bearing waste rocks enriched with Zn-Cu-Cd from the Faro Mine Complex in Canada (Pakostova et al., 2020), streams impacted by polymetallic pollution (Zn, Cd, and Te) in the runoff from an abandoned gold mine in Argentina (Bonilla et al., 2018). It is generally believed that high concentrations of heavy metals diminish microbial diversity and restrict the flourishing of microbial communities due to their toxicity (Yang et al., 2007; Pan et al., 2021). However, some microbes were also observed to be significantly and positively correlated with the concentration of heavy metals and demonstrated tolerance towards such metals, such as *Acidithiobacillus*, *Acidiphilium*, and *Leptospirillum* (Baker and Banfield, 2003; Méndez-García et al., 2015; Chen et al., 2016; Mesa et al., 2017). This phenomenon may be attributed to the evolution of metal resistance genes in these bacteria, enabling them to adapt to elevated metal concentrations by either transforming heavy metals into less toxic forms or pump them out of the cells (Pal et al., 2022). Furthermore, the growth of acidophiles at elevated metal concentrations may be due to an inherent tolerance resulting from their living environment, including metal tolerance through the complexation of free metals by sulfate ions and passive tolerance to metal influx facilitated by an internal positive cytoplasmic transmembrane potential (Dopson et al., 2003, 2014). The pH can also have a significant impact on the tolerance of acidophilic bacteria to metals. Falagán and Johnson (2018) confirmed that some acidophilic bacteria were invariably much more tolerant at pH 2.0 than at pH 3.0. These metal-resistant taxa discovered in AMD sites may have significant potentials for bioleaching, as microbes are the main contributors to the bioleaching of low-grade ores (Roberto and Schippers, 2022; Vera et al., 2022).

Although previous studies have provided valuable insights into the interactions between heavy metals (e.g., Cu, Cd, Pb, Zn) and microbes in AMD, little attention has been paid to uranium (Schippers et al., 1995; Zammit et al., 2014; Kaksonen et al., 2020; Rosendahl et al., 2023), which has both heavy metal toxicity and radioactivity. At the strong acidity and high redox potential of AMD environment, uranium occurs predominantly in the more soluble hexavalent state (VI) in the form of uranyl sulfate complexes (Guerrero et al., 2023), resulting in its release into environmental water bodies and subsequent impact on microbial communities. An exposure experiment made by Zeng et al. (2020) observed that short-term exposure to low concentrations of uranium resulted in a decrease in bacterial richness but an increase in diversity and the presence of uranium induced significant alterations in the community composition and abundance of bacteria, as evidenced by a decrease in the abundance of Proteobacteria and an increase in the abundance of Firmicutes and Bacteroidetes. Recently, some researchers have reported the microbial response to uranium pollution in the vicinity of uranium mines, and a number of uranium-tolerant taxa have been identified. For example, the abundance of *Geobacter* in the anoxic hypolimnion of a former uranium mine open pit in southwestern Sweden may be related to relatively high uranium concentrations (Edberg et al., 2012). Sequencing of microbial communities in soils with different contamination gradients (2–900 mg/kg) from the Ranger uranium mine in northern Australia suggested that bacterial OTUs closely related to *Kitasatospora* spp., *Sphingobacteria* spp., and *Rhodobium* spp. were only present at higher uranium concentrations (Mumtaz et al., 2018). Xiao et al. (2019) found a significant positive correlation between the abundance of *Sphingomonas* sp. and *Pseudomonas* sp. and non-residual uranium concentration in uranium mine contaminated soil of Sichuan province, China. As mentioned above, the research objectives of previous studies were either for contaminated soil (Mumtaz et al., 2018; Xiao et al., 2019; Li et al., 2024), or aqueous environments that are predominantly neutral, weakly acidic, or reducing (Edberg et al., 2012; von Gunten et al., 2018; Zhu et al., 2023), all of which had significant differences from typical AMD environments. High concentrations of uranium may have a direct effect on the microbial community in AMD, but this effect is currently poorly understood. In addition, the role of microbes in the biogeochemical release of uranium in AMD environments is not well-documented, yet it is essential for successfully developing prevention and remediation strategies for uranium- rich AMD.

Stone coal is a combustible, low-heat value, high-rank sedimentary rock mainly derived from early Paleozoic bacteria and algae after saponification and coalification in a marine influenced environment (Dai et al., 2017). It is an important uranium resource (carbonaceous siliceous mudstone type) in southern China (Wang et al., 2018), widely distributed in 10 provinces of southern China, with a total reserve of 61876.7 Mt. Essentially, it represents a kind of early Paleozoic (and in a few cases Permian) pyrite-rich combustible black shale (Dai et al., 2017). With the adjustment of national policies, stone coal mines with low calorific value and severe environmental pollution have gradually been closed. These abandoned mines could generate uranium-rich AMD under weathering for a long time, thereby contaminating soil, surface water, and organisms around the mining area (Xu et al., 2018; Wei and Li, 2023). Abandoned stone coal mines provide an ideal site for understanding the structure of microbial communities in uranium-rich AMD and the mechanisms by which these microbes facilitate the release of uranium.

**Figure 1:**
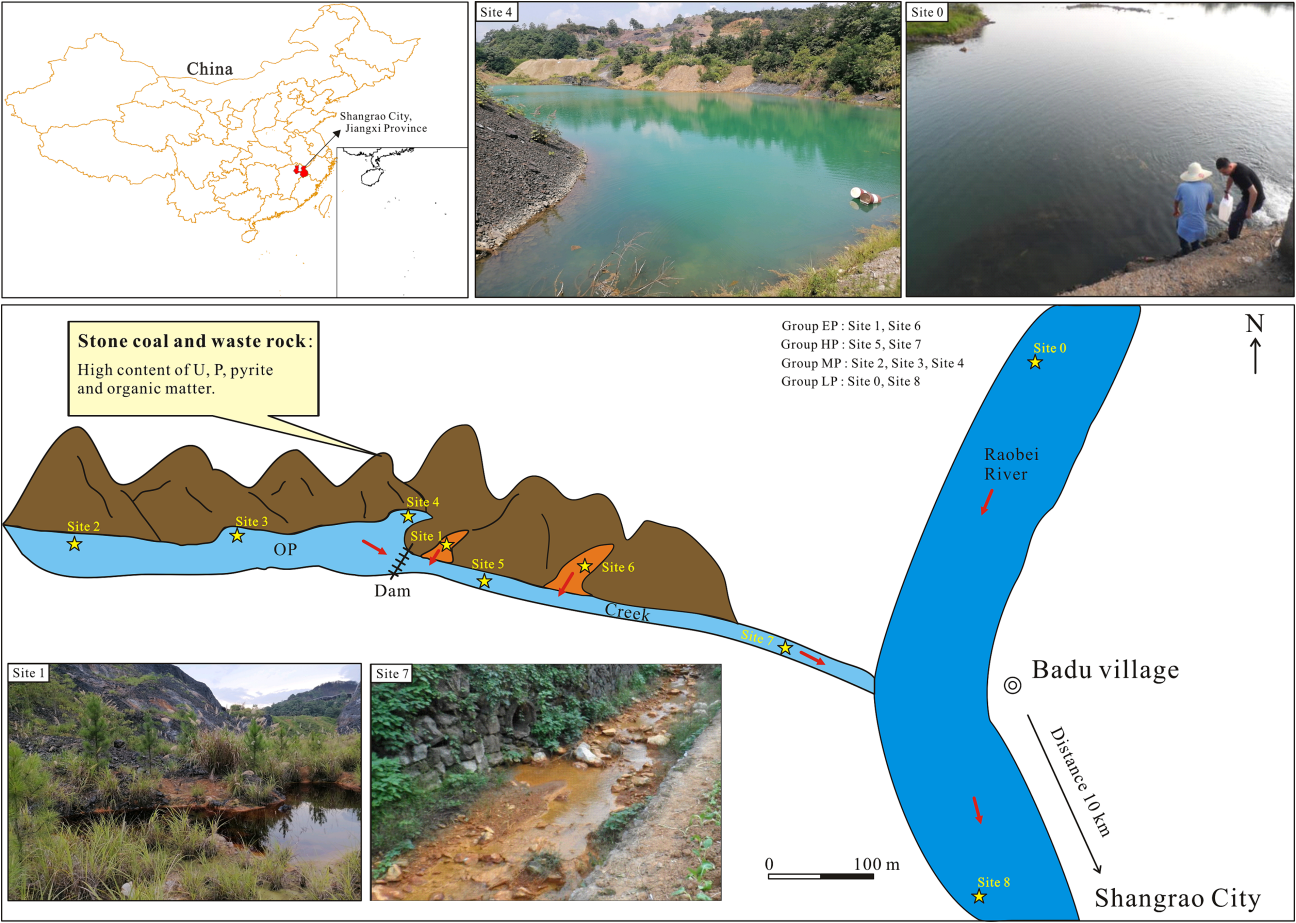
Locations and representative field photos of sampling sites in Badu stone coal mining, AMD-contaminated Creek, and Raobei River in Shangrao City, Jiangxi Province, China. The orange areas represent the newly formed pits due to the mine restoration project. OP, Old pit; EP, extreme pollution; HP, high pollution; MP, moderate pollution; LP, light pollution.

In this study, we focused on the Ba Du stone coal mine area in Shangrao, Jiangxi Province, China, which is a typical area comprising stone coal, with 108.4 Mt. of stone coal reserves and a high degree of exploitation and utilization (Guo, 1990). The mine had been closed for over a decade. The remains of waste rock were then deposited in the mining area, with an area of over 100 ha, where today it can pose a potential environmental hazard due to exposure to air and water (Figure 1, field photo of Site 4). Given the potential for seasonal fluctuations in the microbial community within AMD environments (Hao et al., 2017; Xin et al., 2021), we collected samples of pit water, creek water, and river water in summer (July) and winter (December), respectively (Figure 1), to characterize the temporal and spatial variations of environmental factors and bacterial communities. The research objectives of this study are to (1) reveal the spatial and temporal variations of environmental factors and bacterial diversity and community structure in uranium-rich AMD site, (2) investigate the correlation between bacterial community structure and environmental factors, and (3) identify taxa that are closely related to the biogeochemical release of uranium. This research enhances our understanding of microbial community composition in uranium-enriched AMD environments and its role in the biological mobilization of uranium.

## 2 Materials and methods

### 2.1 Study area and samples collection

The Badu stone coal mine (28°34′3.36″, 117°58′44.83″) is located in the Rao Bei River basin in northeastern Jiangxi Province, approximately 10 km from Shangrao City (Figure 1). The study area has a subtropical monsoon humid climate, with an altitude of 65–331 m, an average temperature of 17.8°C, an average annual precipitation of 1,954 mm, and strong chemical and biological weathering. The Lower Cambrian Hetang Formation is the stratum that hosts stone coal, with an average uranium concentration of 100.64 mg/kg (Yang et al., 2022). Figure 1 shows two types of pits: old pits (open for over 30 years) and new pits (formed from mine restoration projects, around 3 years old). Water from these pits flows into the Creek, then into the Rao Bei River. Based on the sampling locations and varying levels of pH, the sampling sites were divided into four pollution groups, namely extreme pollution (EP, pH < 3), high pollution (HP, 3 ≤ pH < 7), moderate pollution (MP, 7 ≤ pH < 7.5) and light pollution (LP, pH ≥ 7.5). Due to the fact that water samples from each pollution group have various origins and the physicochemical parameters of the water samples vary with seasons, we modified the classifications of specific samples to more realistically and reasonably represent their pollution level, including W5, S3, and W2 (Figure 1; Table 1). A total of 18 water samples were collected in July 2023 (summer) and December 2023 (winter), and were named as S0–S8 and W0–W8, respectively.

**Table 1:** Physicochemical characteristics of all water samples.

Groups	Samples	T	pH	Eh	EC	Na	Mg	K	Ca	Fe	U	SO_4_^2−^	HCO_3_^−^	Cl^−^	NO_3_^−^	TP
Extreme pollution (EP)	S1	31.8	2.84	450	3,830	0.292	111	0.733	21.5	193	390	3,002	bdl	85.18	0.913	2.26
	S6	29.8	2.88	474	5,590	0.626	144	0.425	47.8	400	8,690	5,832	bdl	20.84	bdl	6.54
	W1	12.7	2.38	448	4,270	1.57	135	1.73	72.8	208	920	3,158	bdl	100.8	1.26	6.06
	W6	13.3	2.84	510	5,880	1.32	156	0.912	66.3	454	9,370	6,162	bdl	34.71	0.320	7.04
Heavy pollution (HP)	S5	28.4	6.66	206	1,878	50.3	22.2	7.21	28.6	5.05	49.7	1,077	48	72.11	2.28	0.202
	S7	29.7	5.63	251	2,136	47.2	25.8	6.61	28.9	18.5	404	1,276	47	123.3	1.40	0.422
	W5	12.4	7.29	236	1,962	59.1	43.0	8.06	51.8	5.21	97.0	1,264	56	93.76	2.44	0.308
	W7	12.5	5.45	301	2,230	58.5	36.9	8.10	60.2	22.5	909	1,338	26	126.4	1.40	0.568
Moderate pollution (MP)	S2	30.4	7.38	247	1,538	53.9	19.4	7.43	19.8	0.385	38.8	853	50	46.61	2.28	0.011
	S3	30.1	6.93	229	1,306	52.6	22.5	4.05	17.2	0.228	19.3	689	35	60.53	0.535	0.007
	S4	31.2	7.05	270	1,464	53.7	28.6	3.27	23.3	0.288	46.0	980	47	27.81	0.937	0.013
	W2	12.9	6.69	162	1,783	62.4	28.5	8.71	30.9	0.823	120	1,536	49	71.28	1.75	0.051
	W3	13.1	7.40	243	1,498	63.3	57.9	6.11	38.9	0.872	17.0	1,254	46	70.14	1.74	0.055
	W4	13.2	7.17	243	1,811	60.3	71.2	4.28	46.5	0.823	104	1,823	36	57.42	1.58	0.072
Light pollution (LP)	S0	28.4	7.77	256	291	1.79	2.00	1.10	1.55	0.116	1.25	11.1	55	51.40	4.11	0.031
	S8	28.5	7.38	213	351	2.32	2.23	1.26	1.90	0.188	1.28	9.30	51	50.07	3.56	0.029
	W0	12.4	7.77	227	326	1.97	2.12	1.76	2.50	0.173	2.02	23.4	53	72.39	4.71	0.032
	W8	12.8	7.69	166	413	3.12	4.59	5.80	9.90	0.487	2.93	23.9	47	83.16	4.26	0.086

The values of T, Eh, EC, and U are presented as °C, mV, μs/cm, and μg/L, respectively. pH is no unit. Other parameters are mg/L. Bdl refers to below detectable limit. TP, total phosphorus; T, temperature; EC, conductivity; EP, extreme pollution; HP, high pollution; MP, moderate pollution; LP, light pollution.

Samples were sampled at a depth of approximately10 cm at each site 2 m offshore according to Liang et al. (2017). Three parallel subsamples were collected at each sampling location. Field measurements of water samples, including pH, Eh, electrical conductivity (EC), and temperature (T) were done at three parallel points in each sampling location using a portable multi-parameter water quality meter (YSI ProPlus). The collected samples were divided into two portions for further analysis. One portion was filtered through 0.45 μm membrane and stored in two 500 mL clean polyethylene bottles soaked with 1:1 HNO_3_ beforehand, one of which was acidified with nitric acid to pH < 2 for testing of the major cations and metallic elements, and the other for the analysis of anions. The second portion was passed through 0.22 μm membrane using a vacuum pump, and the resulting precipitate on the membrane was promptly stored in a pre-sterilized centrifuge tube for DNA extraction. All samples were stored in boxes with dry ice and promptly transported to the laboratory.

### 2.2 Geochemical analysis of water samples

The three subsamples were mixed together before geochemical analysis. The concentrations of major cations (Na^+^, K^+^, Mg^2+^, Ca^2+^) and metal elements (Fe and U) were analyzed using inductively coupled plasma mass spectrometry (ICP-MS, LabMS 3000). Anion analysis for Cl^−^, NO_3_^−^, and SO_4_^2−^ was determined by ion chromatography (IC, Swiss Metrohm 881). The concentration of total phosphorus (TP) was measured using a UV–visible spectrophotometer (UV, 1800PC). The results for the concentration of HCO_3_^−^ were obtained by titration.

### 2.3 DNA extraction and PCR amplification

Total microbial genomic DNA was extracted from the precipitates on the membranes of three parallel samples using the E.Z.N.A.^®^ soil DNA Kit (Omega Bio-tek, Norcross, GA, United States) according to manufacturers instructions. The quality and concentration of DNA were determined by 1.0% agarose gel electrophoresis and a NanoDrop^®^ ND-2000 spectrophotometer (Thermo Scientific Inc., United States) and kept at −80°C prior to further use. The hypervariable region V3-V4 of the bacterial 16S rRNA gene was amplified with primer pairs 338F (5′-ACTCCTACGGGAGGCAGCAG-3′) and 806R (5′-GGACTACHVGGGTWTCTAAT-3′) (Liu et al., 2016) by an ABI GeneAmp^®^ 9700 PCR thermocycler (ABI, CA, United States). The PCR reaction mixture included 4 μL 5 × Fast Pfu buffer, 2 μL 2.5 mM dNTPs, 0.8 μL of each primer (5 μM), 0.4 μL Fast Pfu polymerase, 10 ng of template DNA, and ddH_2_O to a final volume of 20 μL. PCR amplification cycling conditions were as follows: initial denaturation at 95°C for 3 min, followed by 27 cycles of denaturing at 95°C for 30 s, annealing at 55°C for 30 s and extension at 72°C for 45 s, and single extension at 72°C for 10 min, and end at 4°C. The three parallel subsamples collected at each sampling location were amplified, and each subsample was amplified in triplicate. The PCR products of these three subsamples were pooled and then extracted from 2% agarose gel. Subsequently, they were purified using the AxyPrep DNA Gel Extraction Kit (Axygen Biosciences, Union City, CA, United States) according to manufacturers instructions and quantified using Quantus^™^ Fluorometer (Promega, United States).

### 2.4 16S rRNA sequencing and data processing

Purified amplicons were pooled in equimolar amounts and paired-end sequenced on an Illumina MiSeq PE300 platform (Illumina, San Diego, United States) according to the standard protocols by Majorbio Bio-Pharm Technology Co. Ltd. (Shanghai, China). Raw FASTQ files were de-multiplexed using an in-house perl script, and then quality-filtered by fastp version 0.19.6 and merged by FLASH version 1.2.7 (Magoc and Salzberg, 2011). Then the optimized sequences were clustered into operational taxonomic units (OTU) using UPARSE 7.1 with 97% sequence similarity level (Edgar, 2013). The most abundant sequence for each OTU was selected as a representative sequence. To minimize the effects of sequencing depth on alpha and beta diversity measure, the number of 16S rRNA gene sequences from each sample was rarefied. The taxonomy of each OTU representative sequence was analyzed by RDP Classifier version 2.2 against the 16S rRNA gene database (Silva v138) using confidence threshold of 0.7.

### 2.5 Statistical analysis

Bioinformatic analysis of samples was carried out using the Majorbio Cloud platform^1^ and R software packages.^2^ Based on the OTU information, rarefaction curves and alpha diversity, such as Chao, Simpson, Shannon index and Coverage were calculated with Mothur v1.48.0 (Schloss et al., 2009). Student’s *T* test and Kruskal-Wallis rank-sum test were used to analyze the inter-group difference of alpha diversity. The canonical correlation analysis (CCA) was performed to investigate the effect of physicochemical parameters on the structure of bacterial community in AMD, and to quantify this effect in conjunction with the Mantel test.

## 3 Results

### 3.1 Physicochemical characteristics of water samples

The water samples showed a wide range of physicochemical gradients (Table 1; Supplementary Table S1). The temperature of summer samples was significantly higher, ranging from 28.4 to 31.8°C, compared to the winter samples which ranged from 12.4 to 13.1°C. In summer, the pH was the lowest in group EP, at 2.86 ± 0.03 (mean ± standard deviation) and the highest in group LP, at 7.58 ± 0.28. Group EP exhibited highest Eh and EC, with average values of 462 ± 17 mV and 4,710 ± 1,245 μs/cm, respectively. The most abundant metal ion in group EP was Fe (297 ± 146 mg/L) and the concentration of Fe decreased sharply from group EP to LP. The variation of other major cations such as Mg (128 ± 23 mg/L in group EP, 2.12 ± 0.16 mg/L in group LP), Ca (34.7 ± 18.6 mg/L in group EP, 1.73 ± 0.25 mg/L in group LP), showed a similar trend as Fe. The concentrations of Na and K were low in the group EP. There was a noticeable decrease in uranium concentration from highly polluted areas to downstream samples, with levels ranging from 390 to 8,690 μg/L in group EP and 49.7–404 μg/L in group HP to 1.25–1.28 μg/L in group LP.

The major anion (SO_4_^2−^) also showed a similar variation trend as Fe. The concentration of SO_4_^2−^ reached the highest value of 4,417 ± 2,001 mg/L in group EP, then dropped to 1,177 ± 141 mg/L in group HP, and reached the lowest value of 10.18 ± 1.24 mg/L in group LP. Due to the strong acidity of group EP (pH < 3), HCO_3_^−^ was not detected, but it was the predominant anion in group LP, at 53 ± 3 mg/L. In addition to sulfate, chloride ion was the most important anion in these four groups, and the concentration range was narrow. The concentration of nitrate in group EP was low (bdl–0.913 mg/L), and increased with distance to the mine, reaching up to 3.834 ± 0.392 mg/L in group LP. Interestingly, total phosphorus in group EP reached as high as 4.40 ± 3.03 mg/L, and its concentration was more than 100 times that of group LP that can receive anthropogenic phosphorus input (Mallin and Cahoon, 2020). Highly elevated P concentrations (P_2_O_5_ generally >0.1%, Dai et al., 2017) in stone coal may contribute to the observed extremely high concentration of total phosphorus.

In general, the main ions occurred in slightly higher concentrations in winter than in summer. For example, the concentration of SO_4_^2−^ was 5,832 mg/L in S6, and reached a higher value of 6,162 mg/L in W6. It is noteworthy that a notable seasonal variation in water chemistry parameters at Site 1 was observed, particularly evident in the decline in pH during the winter sampling. This phenomenon can be attributed to the limited size of the pit watershed at Site 1, which experiences prolonged evaporation and elevated temperatures during the summer and fall. These conditions expedite the oxidation of mine wastes, leading to increased acidity levels, as discussed by Hao et al. (2017) and Xin et al. (2021).

### 3.2 Bacterial diversity

A total of 1,358,234 quality sequences (average sequence length, 409–424 bp) were obtained from 18 samples (9 samples per season), of which the number of sequences per sample ranged from 58,733 to 93,078 (Table 2). The Goods coverage values (0.9860–0.9994) suggested that the level of analysis was sufficient to characterize bacterial communities in all samples, which was consistent with the result from rarefaction curves (Supplementary Figure S1A). The indices of community diversity, such as OTU, Shannon, and Simpson, revealed that the variations of bacterial diversity among different groups were greater than the seasons. In summer, the samples of group HP had the highest diversity, with OTU numbers of 2,447–2,894, followed by the group MP (OTU, 612–1,016) and group LP (626–778). The bacterial diversity in group EP (the two most acidic samples, S1 and S6) was lowest, with OTU numbers of 253–252. The Rank- abundance curve based on OTU classification level also clearly showed that the bacterial richness and evenness of group EP was the lowest, and that of group HP was the highest, followed by groups MP and LP (Supplementary Figure S1B). The group HP exhibited the highest bacterial diversity and abundance, rather than the group LP with the lowest pollution level, similar to the observations in the Aha Watershed made by Sun et al. (2015). On the one hand, this may be related to the fact that the water body in group HP is rich in nutrients such as nitrate and phosphorus (Table 1). On the other hand, as shown in Figure 1, the water body in the group HP receives the effluents from groups EP and MP, and the structure of the bacterial community is also characterized by a mixture of them (see the description below).

**Table 2:** Results of Illumina MiSeq high-throughput sequencing (16S rRNA) and alpha diversity indices.

Groups	Samples	Sequences	Mean length	OTU	Shannon	Simpson	Ace	Chao	Coverage
Extreme pollution (EP)	S1	76,695	424	253	2.32	0.205	328	343	0.9986
	S6	80,690	423	252	2.65	0.136	342	356	0.9985
	W1	63,354	424	217	2.53	0.166	234	232	0.9994
	W6	58,733	424	469	2.19	0.303	556	532	0.9980
Heavy pollution (HP)	S5	80,010	420	2,447	5.66	0.021	2,687	2,696	0.9919
	S7	86,596	418	2,894	5.73	0.024	3,272	3,242	0.9886
	W5	71,171	416	3,860	6.75	0.004	4,304	4,211	0.9860
	W7	61,058	421	937	2.87	0.258	1,035	1,016	0.9969
Moderate pollution (MP)	S2	93,078	416	1,016	3.72	0.063	1,279	1,212	0.9943
	S3	86,633	414	612	3.21	0.084	900	798	0.9954
	S4	80,091	412	699	3.19	0.092	896	865	0.9961
	W2	69,688	409	478	3.36	0.077	576	550	0.9976
	W3	79,987	413	1,050	3.31	0.089	1,387	1,305	0.9931
	W4	65,660	416	1,439	4.02	0.059	1,747	1,678	0.9926
Light pollution (LP)	S0	86,985	420	778	3.78	0.049	1,046	946	0.9952
	S8	84,746	423	626	3.37	0.079	852	786	0.9961
	W0	66,547	424	809	3.56	0.075	1,009	934	0.9956
	W8	66,512	421	1,058	3.87	0.063	1,270	1,193	0.9947

OTU, operational taxonomic units; EP, extreme pollution; HP, high pollution; MP, moderate pollution; LP, light pollution.

All these results suggest that an extremely acidic environment was likely to reduce microbial diversity, and similar results had been observed in other AMD environments (Sun et al., 2019; Zhang et al., 2019; Chen et al., 2021). The results of statistical analysis revealed that there were no significant differences in Shannon index between samples from summer and winter, while there were distinct differences between the four groups, especially between the groups EP and HP (*p* < 0.01) and between the groups MP and HP (*p* < 0.05) (Supplementary Figures S1C,D). Bacterial diversity may be primarily affected by the pollution groups rather than seasonal variations.

### 3.3 Bacterial community composition

**Figure 2:**
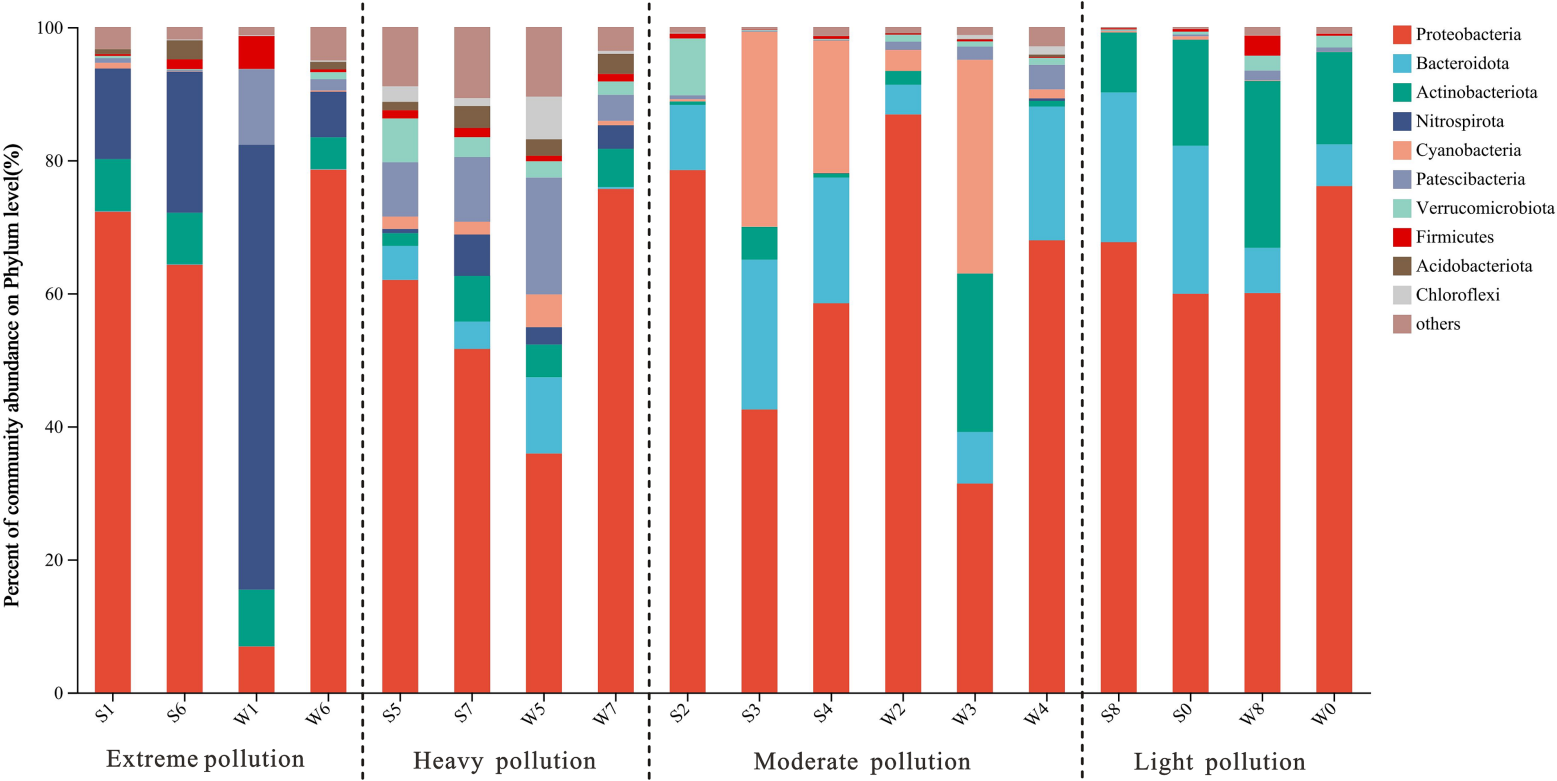
Microbial community structure in water samples at phylum level, which showed the abundances of the top 10 phyla along the pollution groups.

All classifiable sequences were ultimately clustered into 49 phyla. Proteobacteria was the most abundant phylum in all samples (59.8 ± 20.0%), except W1 and W3, which had the highest relative abundance of Nitrospirota (66.9%) and Cyanobacteria (32.1%), respectively (Figure 2). This is consistent with previous reports that Proteobacteria were dominant in AMD ecosystems (Bonilla et al., 2018; Pakostova et al., 2020). Other phyla such as Bacteroidota, Actinobacteriota, Nitrospirota, and Cyanobacteria were also detected, but the proportion of these phyla differed among four groups. The relative abundances of Bacteroidota increased with decreasing pollution levels, with abundance of 0% in group EP but 14.5 ± 9.2% in group LP. In contrast, Nitrospirota was an important member of the group EP (6.8–66.9%), but was nearly undetectable in the groups MP and LP. The most significant seasonal differences occurred at Site 1. Proteobacteria was dominant in summer and accounted for 72.2% of all sequences (S1), while Nitrospirota mainly occurred in winter, with the abundance of 66.9% (W1). The observed variations in seasonal patterns at Site 1, particularly at the phylum level, may be attributed to the presence of distinct genera (see discussion section below).

**Figure 3:**
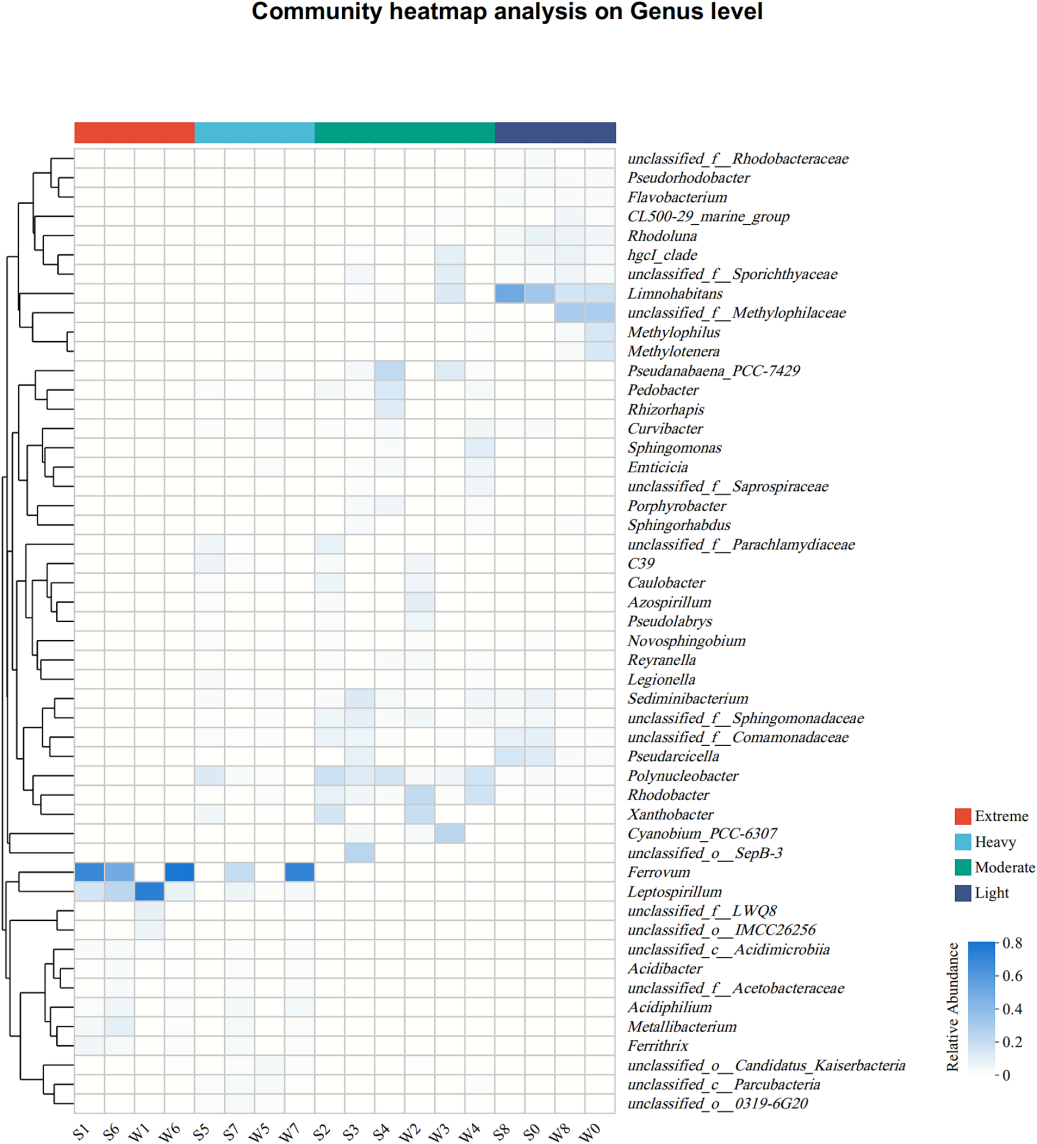
Heat map of the dominant genera (top 50 genera in total abundance) along the pollution groups. The relative abundances (ranging from 0 to 1) of genera are depicted by the color intensity.

At the genus level, the differences in bacterial community compositions between groups remained more pronounced than between seasons (Figure 3; Supplementary Figure S2). In group EP, *Ferrovum* was the most dominant member in S1, S6, and W6, with abundance of 62.1, 44.2, and 72.1%, respectively, but was hardly observed in W1. *Leptospirillum* was another abundant genus with abundance over 5% in all samples from group EP, especially in W1 (66.9%). Both of them were categorized as chemoautotrophic iron-oxidizing bacteria that were widespread in other AMD sites (Baker and Banfield, 2003; Chen et al., 2016; Sun et al., 2019). In addition, heterotrophic acidophilic bacteria were found in group EP, such as *Metallibacterium* (3.3 ± 3.8%), *Ferrithrix* (2.2 ± 2.1%), and *Acidiphilium* (2.0 ± 1.9%). Similar to group EP, *Ferrovum* and *Leptospirillum* were also important members at Site 7 (a sampling site in the group HP), which was strongly polluted by highly acidic AMD (Site 6, pH < 3). However, some neutrophilic bacteria, such as *Xanthobacter* and *Polynucleobacter,* were observed at Site 5, mainly due to the fact that Site 5 is primarily recharged from the old pits. The main genera of the group MP were completely different from those of the group EP, although both of them were collected from the pits of stone coal mine. *Polynucleobacter*, *Rhodobacter*, *Xanthobacter*, *Pseudanabaena_*PCC-7429, and *Sediminibacterium* were enriched in group MP. *Pseudanabaena_*PCC-7429 at Site 4 was dominant in summer (S4, 19.6%) but was scarce in winter (W4, less than 1%). *Limnohabitans* was a major member in group LP, especially in summer (29.7–45.1%). Due to river self-purification and natural attenuation, the downstream bacterial community (Site 8) had returned to a level similar to upstream background level (Site 0), as proposed by He et al. (2019).

In summary, bacterial communities in uranium-rich AMD were similar to typical AMD environments, with noticeable shifts along pollution groups. Each group had distinct characteristics: EP had more acidophilic chemolithotrophic bacteria; MP was rich in heterotrophic bacteria with higher optimum growth pH; HP was characterized by a mix of both; LP was dominated by neutrophilic heterotrophic bacteria, such as *Limnohabitans* and *Pseudarcicella*, which were widely detected in freshwater ecosystems (Hahn et al., 2010; Guo et al., 2021).

### 3.4 Relationship between bacterial community and environmental conditions

**Figure 4:**
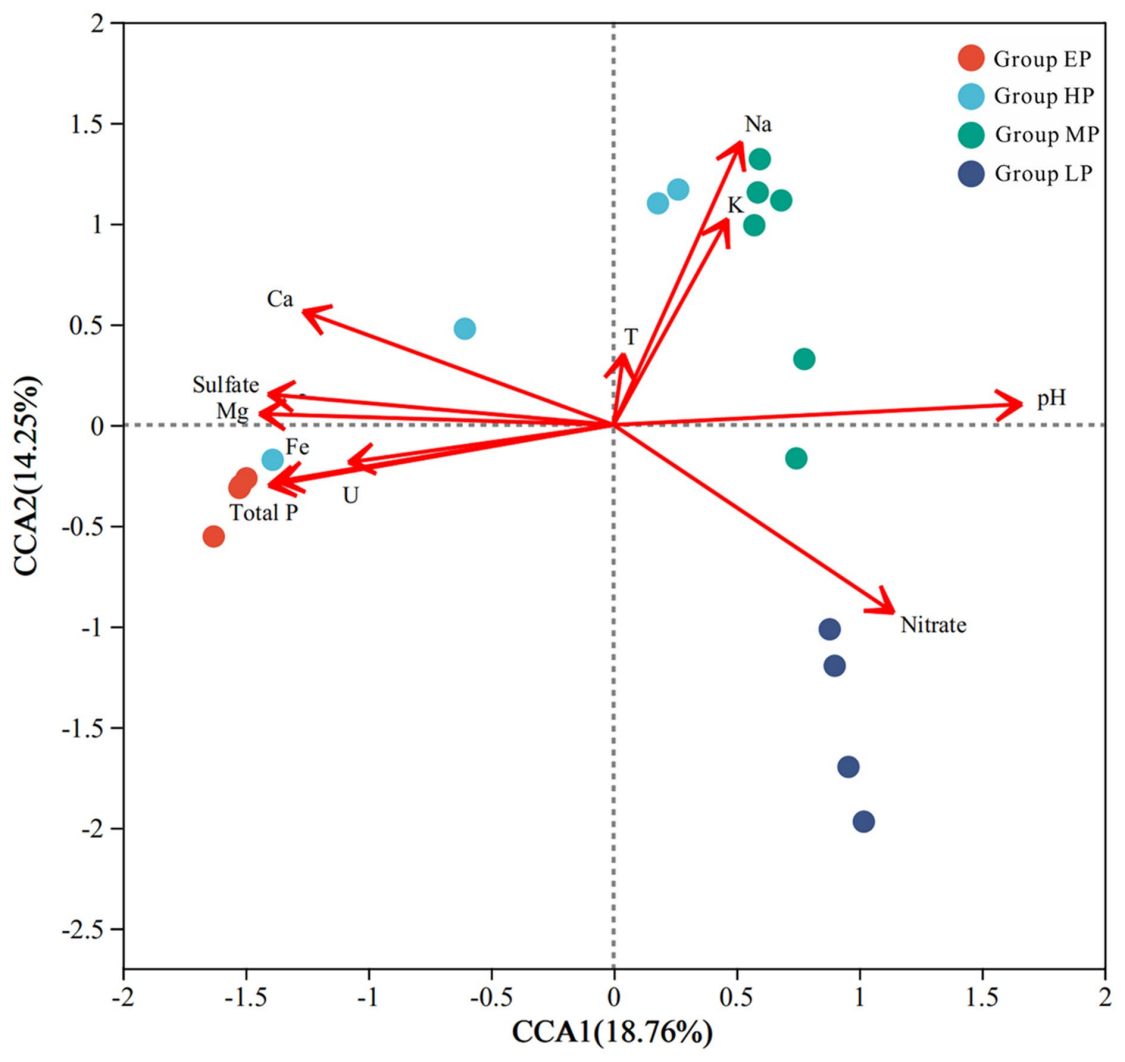
CCA ordination plot showing the relationship between environmental conditions and bacterial communities. The samples from the four groups were represented by circles of different colors. T, temperature.

Based on the result from the Detrended Correspondence Analysis (DCA), which showed that the size of first axis is 7.031 (>3.5), CCA was performed to investigate the relationship between selected physicochemical parameters (pH, T, Na^+^, Mg^2+^, K^+^, Ca^2+^, Fe, U, sulfate, nitrate, and TP) and bacterial communities (Figure 4). CCA axis 1 was positively correlated with pH and negatively correlated with concentrations of main ions and metals. CCA axis 2 had a positive correlation with temperature and concentrations of alkali metals. The bacterial communities of group EP were mainly shaped by pH, TP, Fe and U concentrations, while that of group MP were primarily affected by alkali metals (Na and K) concentrations. Bacterial communities in the group HP still showed mixed influence by groups EP and MP. Nitrate concentration was the environmental factor with the highest explanation value in the group LP. The relatively small magnitude of the temperature vector indicated that temperature was weakly correlated to bacterial community, which further demonstrated that seasonal variation was not the key factor driving microbial communities in this study. Moreover, a Mantel test was conducted to analyse the environmental conditions responsible for shaping the bacterial community (Supplementary Table S2). The results suggested that bacterial communities were closely correlated to U, Fe, TP concentrations and pH, with r values of 0.56, 0.52, 0.51, and 0.49 at *p* ≤ 0.001 level, respectively, but not temperature (*r* = −0.03).

**Figure 5:**
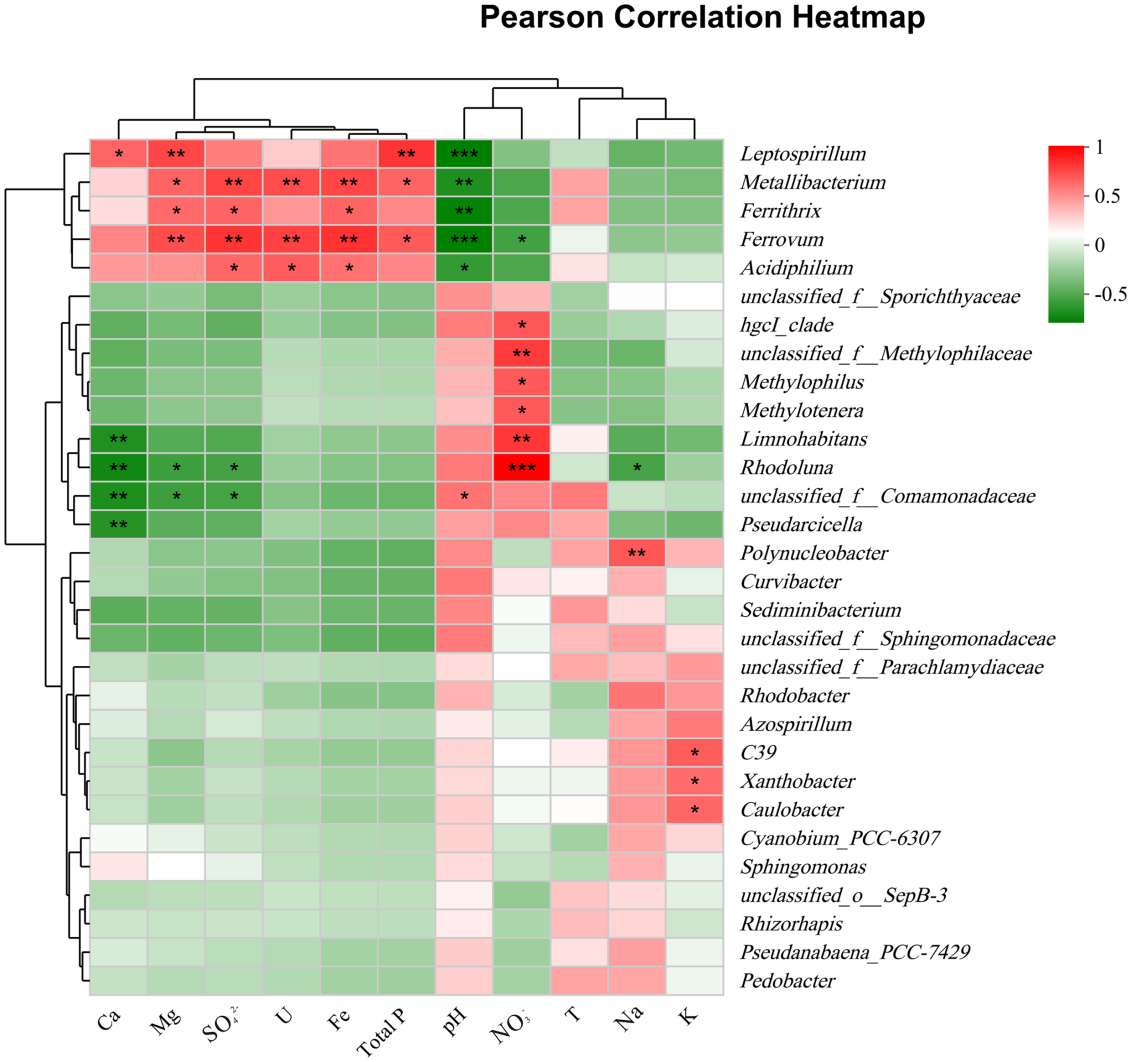
Pearson correlation heatmap between the top 30 genera and important environmental factors. *, 0.01 < *p* ≤ 0.05; **, 0.001 < *p* ≤ 0.01; ***, *p* ≤ 0.001.

As shown in Figure 5, a heatmap was used to illustrate the correlation between bacterial communities (genera level) and important environmental factors. Mg, SO_4_^2−^, Fe, U, and TP concentrations were significantly positively correlated with the abundance of the same genera, especially the acidophilic bacteria *Metallibacterium*, *Acidiphilium*, *Ferrovum*, *Leptospirillum,* and *Ferrithrix*, which showed negative correlation with pH. The above results suggested that AMD ecosystem may facilitate the growth of these taxa. However, a genus affiliated with uncultured Comamonadaceae (unclassified_f_Comamonadaceae), *Curvibacter, Sediminibacterium,* and *Polynucleobacter*, the common members in group MP, showed the opposite relevance to these acidophilic bacteria, which indicated that these taxa favor environments with higher pH. *Rhodoluna* and *Limnohabitans* were strongly positively correlated with nitrate, suggesting a potential relevance to nitrogen cycling. Overall, pollution groups strongly influenced bacterial communities, with pH, U, Fe, TP, Mg, sulfate, and nitrate concentrations being key factors.

## 4 Discussion

### 4.1 Spatial and temporal variations of uranium concentration along the pollution groups

Consistent with findings in other watersheds affected by AMD (Sun et al., 2015; Bonilla et al., 2018; Zhang et al., 2019), this study also discovered that most physicochemical parameters, such as acidity and the concentration of Fe and SO_4_^2−^, showed a decreasing trend along the pollution levels (Table 1; Supplementary Table S1). Particularly notable was the transition in uranium concentration from an extremely high level (9,370 μg/L) in the group EP to a level approaching the global average for major rivers (0.51 μg/L, Chabaux et al., 2003) in the group LP (Supplementary Figure S3). The concentration of uranium in natural water bodies is generally low, with river water typically exhibiting concentrations below 4 μg/L. Instances of elevated uranium concentrations in surface water exceeding mg/L level are uncommon and typically attributed to localized mineralization or evaporation from alkaline lakes (Smedley and Kinniburgh, 2023). In this study, extremely high concentrations of uranium were detected in AMD-contaminated effluents, with a maximum value of 9,370 μg/L, which is about five orders of magnitude above the background value of world rivers, and far exceeds the WHO provisional guideline value of 30 μg/L for drinking water (WHO, 2017). Guerrero et al. (2023) noted the presence of the ^238^U in AMD from an abandoned metal sulfide mine at a concentration of 700 mBq/L (equivalent to approximately 57 μg/L), which was two orders of magnitude of the world river background value. These findings suggested that AMD may also be an important source of uranium in the environment, which has been neglected in the past due to the fact that uranium is a non-sulfophilic element, whereas AMD production is mainly associated with sulfide minerals like pyrite (Acharya and Kharel, 2020).

**Figure 6:**
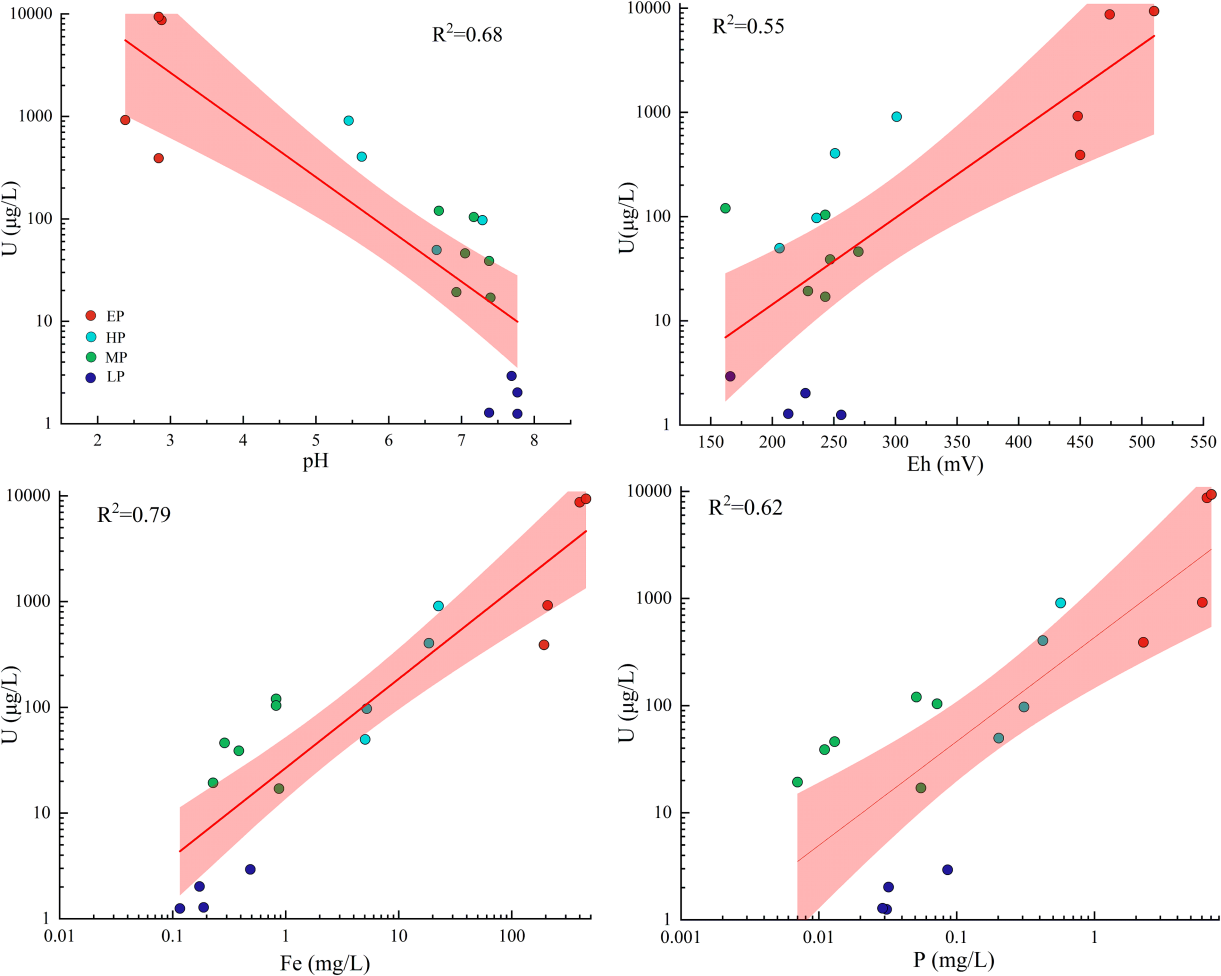
Relationships between the concentrations of uranium and selected physicochemical parameters in water samples. Regression lines and R^2^ are also shown. Shaded areas are the 95% confidence intervals. Different colored dots represent samples collected from different groups.

In the present study, natural attenuation mechanisms play a significant role in the observed variations in uranium levels among distinct groups. Similar occurrences of natural attenuation have been documented in analogous AMD environments, encompassing secondary mineral adsorption and co-precipitation, carbonate mineral buffering, and dilution by non-contaminated water sources (He et al., 2019; Zhang et al., 2019). Specifically, the new pit water (group EP) was the source of AMD contamination, characterized by low pH (pH < 3), high Eh (Eh > 400 mV), and high concentration of SO_4_^2−^. In a typical AMD environment similar to that of this study, uranium predominantly existed in the species of highly soluble uranyl sulfate complexes (VI) (Guerrero et al., 2023). After flowing into the Creek, due to the influence of natural attenuation, the pH of the Creek increased to a range of 5–7. This led to the rapid occurrence of abiotic Fe^2+^ oxidation and Fe^3+^ hydrolysis (Senko et al., 2008), ultimately resulting in the formation of significant quantities of iron precipitation (Figure 1, field photo of Site 7). In addition, as shown in Figure 6, the concentrations of uranium in the water samples were positively correlated with the concentrations of total phosphorus and Fe, with R^2^ values of 0.62 and 0.79, respectively. Therefore, it can be deduced that the decline in uranium concentrations in group EP was primarily due to the co-precipitation of uranium with phosphate and iron hydroxide in Creek resulting from an increase in pH, which was consistent with the finding obtained by Kříbek et al. (2018) and Santofimia et al. (2022). However, it should be noted that the concentrations of uranium in the group HP remained well above the WHO drinking water guidance value (Supplementary Figure S3). After the Creek flowed eastward into the Raobei River, the natural dilution effect further intensified, resulting in a rise in the river’s pH to a range of 7–8. Subsequently, the concentrations of the primary physicochemical parameters returned to levels similar to those upstream, with uranium concentrations ranging from 1.25 to 2.93 μg/L, closely with the average value of worldwide major rivers (Chabaux et al., 2003).

It is worth noting that the physicochemical characteristics (such as pH, Eh, Fe, U, etc.) of the water samples obtained from the old pits (group MP) differed significantly from those of the new pits (group EP), despite both being relatively confined pit water. These differences can likely be attributed to variations in the formation time of the pits and the acid neutralization capacity of the waste rock piles. The formation period of the old pits surpassed 30 years, and following over three decades of oxidation, the acid generation potential of the waste rock has become limited (Xin et al., 2021). Additionally, prior researches have revealed a significant variation in the concentration of carbonate minerals present in stone coal (ranging from 0.45 to 20.0%, Jiang et al., 1994), with the enhanced neutralization capacity of carbonate minerals potentially serving as another crucial factor leading to the near-neutral pH level (7.10 ± 0.27) observed in the water samples of the old pits. The acid-neutralizing capacity of stone coal waste rock has been demonstrated by indoor leaching experiment (Lin et al., 2017). Similar near-neutral pH of AMD-contaminated water due to buffering by carbonate minerals also was observed in several streams contaminated with coal mine AMD on the west coast of South Island, New Zealand (Lear et al., 2009). The new pits, in contrast to the old pits, were formed gradually over a period of approximately 3 years and contain elevated contents of pyrite in the surrounding waste rocks. This led to a robust acid production process catalyzed by acidophilic bacteria (as described below), resulting in a higher acidity in the water within the new pits and a notably increased concentration of uranium (Supplementary Figure S3).

In addition to significant spatial disparities in uranium concentration resulting from pollution groups, there was also a distinct seasonal fluctuation. Specifically, concentrations were found to be slightly higher during the winter compared to the summer, potentially attributed to seasonal precipitation. The predominant rainfall in the study region occurs during summer, leading to dilution and subsequent decrease in dissolved uranium concentration. Similar trends were noted in the variations of other major ions including Mg^2+^, K^+^, Ca^2+^, and SO_4_^2−^, with analogous findings reported in the acidic lake of Nanshan Iron Mine in Anhui Province, China (Hao et al., 2017; Xin et al., 2021).

### 4.2 Potential uranium-tolerant taxa and mechanisms by which they catalyze uranium enrichment in AMD

In the present study, alterations in bacterial diversity and community composition with the pollution groups were in response to shifts in environmental factors. Specifically, the bacterial diversity (OTU) in the new pits (group EP) characterized by extreme AMD pollution was significantly lower compared to other sampling sites (Supplementary Figure S3), which is consistent with results from previous studies on AMD systems (Baker and Banfield, 2003; Lear et al., 2009; Zhang et al., 2019). This suggested that only a limited number of bacterial species can survive in such extreme environments. Harsh environmental conditions, characterized by high acidity and elevated levels of uranium and iron, exerted ecological pressures on bacterial survival (Sun et al., 2015), resulting in the persistence of only those taxa with tolerance (Hesse et al., 2018). The results of statistical heatmap indicated that the abundance of *Ferrovum*, *Acidiphilium*, and *Metallibacterium* increased with increasing uranium concentration (Figure 5). These bacteria were significantly positively correlated with uranium concentration (*p* ≤ 0.05), consistent with bacteria identified in ecosystems surrounding uranium mines, including *Geobacter*, *Kitasatospora*, and *Sphingomonas* (Edberg et al., 2012; Mumtaz et al., 2018; Xiao et al., 2019), indicating that they may all possess potential uranium tolerance. Dopson et al. (2014) proposed that acidophiles confront high metal loads through abiotic and biotic mechanisms, of which precluding the entry of free metal ions into the cell due to complexation of free metals by sulfate ions is one of the important mechanisms. In strongly acidic AMD environments, uranium also forms complexes mainly with sulfate ions (Guerrero et al., 2023). It is hypothesized that this mechanism may also contribute to the tolerance of acidophiles to uranium, although it need to be further revealed.

*Ferrovum*, a chemolithotrophic iron-oxidizing bacterium, was dominant in the group EP with the highest uranium concentration (except W1). The optimum growth pH of the type species *Ferrovum myxofaciens* is 3.0 (Johnson et al., 2014), which is similar to the group EP. The distribution of microbial communities in AMD at the regional scale in southern China has confirmed that *Ferrovum* is widespread at AMD sites and dominant under moderate pH conditions (pH > 2.4) (Kuang et al., 2013), which is consistent with the results of the present study.

*Leptospirillum*, the more acid-tolerant member, was another important acidophilic iron-oxidizing bacterium of the group EP. It is primarily detected in extremely acidic AMD sites (Schrenk et al., 1998; González-Toril et al., 2003; Chen et al., 2013; Pakostova et al., 2020) and is a important contributor to the production of AMD (Coram and Rawlings, 2002; Baker and Banfield, 2003; Bonilla et al., 2018; Huang et al., 2021). Furthermore, although the correlation between *Leptospirillum* abundance and uranium concentration was not that significant (Spearson’r = 0.23, Figure 5), it was the dominant taxon in the winter sample of Site 1 (W1, U = 920 μg/L), with an abundance of 66.9% (Figure 3), also indicating its involvement in facilitating uranium release. Likewise, *Leptospirillum* is one of the acidophilic genera used in the bioleaching of uranium ore (Kaksonen et al., 2020), which further confirms its tolerance to high uranium concentrations. As shown in Supplementary Table S3, the distribution of these uranium-resistant bacteria varied across the pollution groups, with the highest abundance observed in the group EP, followed by the group HP, and almost undetectable in the groups MP and LP, suggesting that they were strongly influenced by pH.

Unexpectedly, in terms of relative bacterial abundance, the winter samples of Site 1 exhibited a significant prevalence of *Leptospirillum*, constituting 66.9% of the microbial community, whereas *Ferrovum*, which was predominant in the summer samples, was nearly absent, comprising less than 1% of the community (Figure 3; Supplementary Figure S2). This phenomenon can be attributed to the preference of *Leptospirillum* for lower pH environments (pH < 2.4), while it typically exists as a minor constituent at higher pH levels (Sand et al., 1992; Chen et al., 2016). Additionally, some reports have recorded that *Leptospirillum* exhibits a high tolerance to toxic ions (Yang et al., 2007). Our observations at Site 1 revealed increased acidity (pH = 2.38) and higher uranium concentrations during the winter season compared to the summer season (Table 1). In conditions of energy limitation, the competition between *Leptospirillum* and *Ferrovum* for ferrous ions led to a pronounced rise in relative abundance of *Leptospirillum* in environments characterized by lower pH levels. A similar example was documented in two AMD lakes of Anhui Province, China (Xin et al., 2021). The strong variation in pH value was the key factor driving the significant difference in bacterial communities between summer and winter at Site 1, which is consistent with the results of relevant studies (e.g., Chen et al., 2013; Korehi et al., 2014; Liu et al., 2014). In addition, the results of CCA analysis and Mantel test further supported that pH as the major factor determining microbial community structure (Figure 4; Supplementary Table S2). Such a drastic change in pH was not observed at Site 6, and thus the structure of the bacterial community was similar between the summer and winter samples.

**Figure 7:**
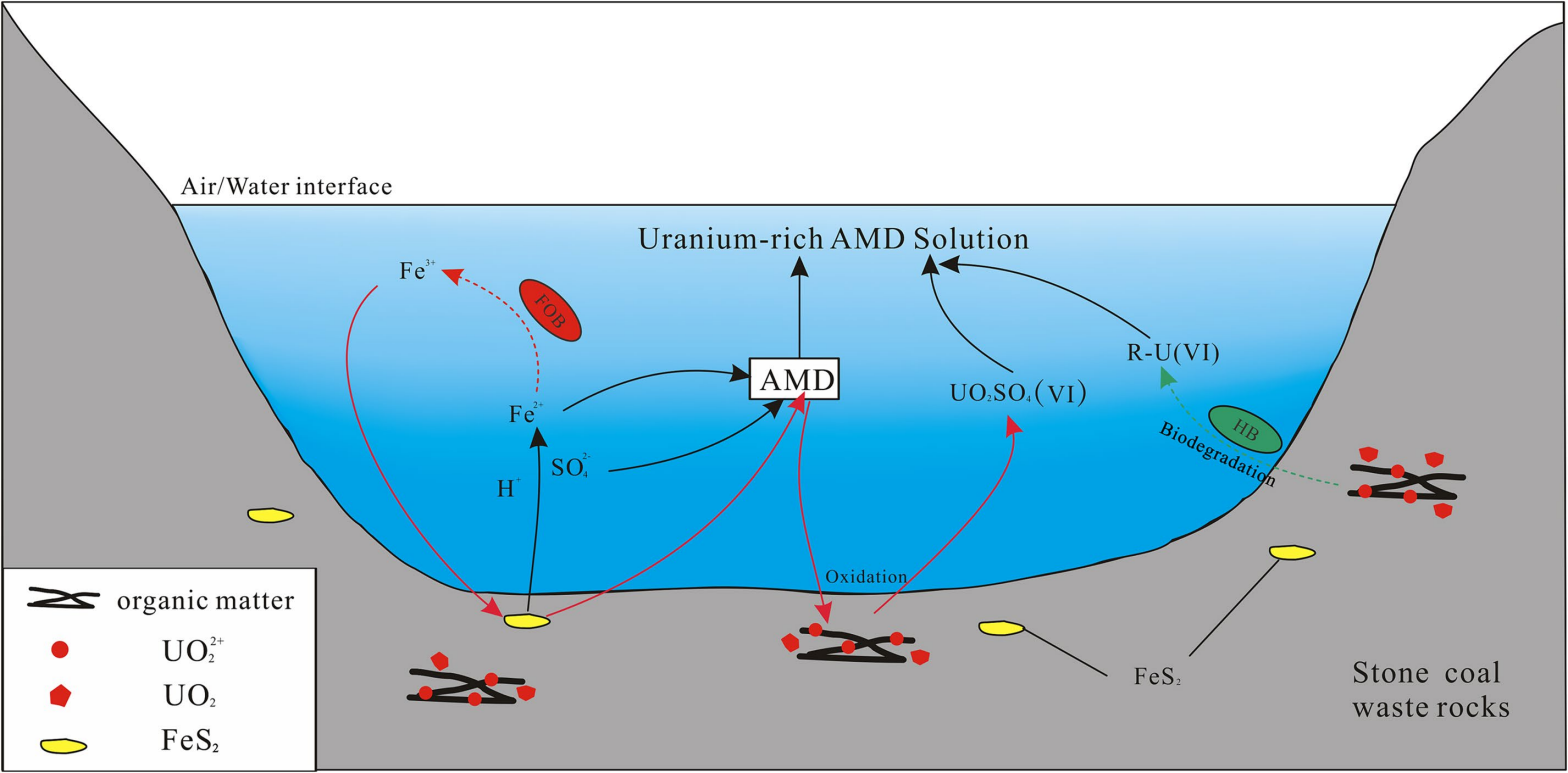
A schematic diagram displaying the biogeochemical release of uranium in the AMD environment of a stone coal mine. The red and green arrows indicate the different release mechanisms of uranium, respectively. The dashed arrow lines represent biological processes in which bacteria are involved, and the solid lines represent abiotic chemical processes. FOB, ferrous iron-oxidizing bacteria; HB, heterotrophic bacteria; R-U, the dissolved uranium bound to organic complexes. UO_2_ and UO_2_^2+^ refer generally to uranium in the form of tetravalent minerals and in the form of hexavalent uranyl ions adsorbed to organic matter from stone coal waste rock, respectively.

The oxidation of ferrous is essential for chemoautotrophic bacteria such as *Ferrovum* and *Leptospirillum* to obtain energy. The primary oxidant in the production of AMD, ferric iron, is mainly obtained by these indigenous acidophilic iron-oxidizing bacteria through the oxidation of ferrous iron, as the abiotic oxidation rate of ferrous iron is slow under acidic conditions (Singer and Stumm, 1970). The generated ferric iron can further oxidize sulfide minerals to produce acid and promote the oxidation and cycling of reduced sulfur (Chen et al., 2016; Vera et al., 2022). AMD generation processes driven by indigenous iron-oxidizing bacteria were usually accompanied by a decrease in pH and an increase in the concentrations in SO_4_^2−^ and Fe (Sun et al., 2015), and similar results also occurred in the present study. The main determining factors for uranium migration include pH, Eh, and the redox state of uranium (IV, VI) (Cumberland et al., 2016). A portion of the uranium present in stone coal was found to be adsorbed onto organic matter in the form of uranyl ions (VI), with the remaining portion existing in the form of tetravalent minerals (such as xenotime and rhabdophane, Wang et al., 2017). In the strongly acidic and oxidizing environments where anions are dominated by SO_4_^2−^, uranium migrates mainly by forming the highly soluble UO_2_SO_4_(VI) (Guerrero et al., 2023). Thus, it can be deduced that the enrichment of uranium in the new pits of the abandoned stone coal mine was primarily influenced by the highly acidic and oxidizing conditions of the AMD environment. Indigenous iron-oxidizing bacteria play an important catalytic role in the formation of AMD, thus indirectly facilitating the release of uranium (Figure 7). With the depletion of sulfide minerals (the energy source of iron-oxidizing bacteria) in the waste rock and the neutralization of carbonate minerals, the pH of the pit water gradually rises, leading to the precipitation of most of the uranium in the form of phosphate and iron hydroxides. The uranium present in the solution undergo a conversion to primarily exist in the low-solubility form of an uranyl carbonate complex, as opposed to uranyl sulfate, due to the results from previous studies indicating that uranium predominantly forms a uranyl carbonate complex (VI) under oxidizing and neutral alkaline conditions (pH = 7–9) (Cumberland et al., 2016; Smedley and Kinniburgh, 2023).

In addition to autotrophic bacteria, uranium-tolerant heterotrophic bacteria, *Acidiphilium* and *Metallibacterium*, also occupied a certain proportion in the groups EP and HP, which are common members of AMD sites (Chen et al., 2016). The synergisms of autotrophic and heterotrophic bacteria is beneficial for optimizing the community structure of AMD microbial systems. Specifically, autotrophs rely on heterotrophs to remove organic matter that is inhibitory for their growth, while heterotrophs utilize the organic matter produced by autotrophs for growth energy, thus driving the carbon cycle (Baker and Banfield, 2003; Schippers et al., 2010; Chen et al., 2016). Furthermore, these two genera also play an important role in driving the iron cycle in the AMD system (Baker and Banfield, 2003; Sun et al., 2015), as some of their species possess the physiological ability to reduce Fe^3+^ to Fe^2+^ (Johnson and Bridge, 2002; Ziegler et al., 2013). This process regenerates the energy source for iron-oxidizing bacteria, and facilitates the flourishing of iron-oxidizing bacteria and the continued production of AMD (Xin et al., 2021). In contrast to metal sulfide mines (e.g., copper ore), waste rocks from stone coal mines not only contain abundant pyrite, but also have a high TOC content (averaging 15–25%, Jiang et al., 1994). A considerable portion of uranium in stone coal was found to be adsorbed onto organic matter in the form of uranyl ions (VI), accounting for about 50% (unpublished data, Wei et al., under review), which was further supported by the significant positive correlation between uranium and TOC (Wang et al., 2017). While direct evidence is currently lacking, it can be hypothesized that heterotrophic bacteria may also facilitate the release of uranium through consuming organic matter in stone coal waste rock (Figure 7). The biodegradation experiments of eight heterotrophic bacteria isolated from the organic-rich Lubin copper mine in Poland have demonstrated that these bacteria can utilize the organic matter present in black shale as their sole carbon and energy source (Matlakowska et al., 2010). The process of biodegradation of organic carbon was also accompanied by the release of toxic heavy metals in the form of organic complexes, with the concentration of leached metals being approximately three times that of sterile control experiments. Xia et al. (2020) further demonstrated through experiments that during the biological oxidation by heterotrophic bacteria, complexation with citric acid was identified as the most important mechanism for facilitating the release of uranium in uranium-rich rhyolite.

## 5 Conclusion

This article investigated the bacterial composition in uranium-rich AMD from an abandoned stone coal mine and the mechanisms for their involvement in facilitating uranium leaching and release. AMD originating from new pits with strong acid production capacity was an important source of uranium pollution in aquatic environments. The formation of differences in uranium contamination was influenced by natural attenuation, differential pit formation time, and variations in acid-neutralizing capacity. Bacterial diversity was lowest under extreme pollution, resembling that of a typical AMD ecosystem, dominated by acidophilic chemolithoautotrophic iron-oxidizing bacteria and acidophilic heterotrophic bacteria. As pollution levels decreased, there was a notable increase in microbial diversity, characterized by a reduction in acidophilic bacteria and an increase in neutrophilic heterotrophic bacteria.

The concentrations of major ions in water samples were slightly lower in summer than in winter, attributed to the intensified summer precipitation. While seasonal fluctuations in microbial community composition were generally insignificant, notable variations were observed at specific sampling locations. Physicochemical parameters, including pH, U, Fe, total phosphorus, Mg^2+^, sulfate, and nitrate concentrations were the key environmental factors affecting the structure of the bacterial community. *Ferrovum*, *Leptospirillum*, *Acidiphilium* and *Metallibacterium* were the dominant acidophilic bacteria. They have been demonstrated to tolerate high concentrations of metals (e.g., Cu, Ni, Zn, Fe) through both biotic and abiotic mechanisms (Dopson et al., 2014). In the present study, they were also found to be positively correlated with uranium concentrations and may be potentially tolerant to uranium, yet the underlying mechanism remains unclear. Additionally, these genera play an important role in contributing to the release of U through a variety of indirect mechanisms. Archaea are also important contributors to acid mine drainage and may have a significant impact on iron and sulfur cycles (Edwards et al., 2000). Therefore, the diversity of archaeal communities in uranium-rich AMD still requires further investigations.

## Data availability statement

The original contributions presented in the study are included in the article/Supplementary material, further inquiries can be directed to the corresponding author.

## Author contributions

XW: Conceptualization, Data curation, Investigation, Software, Writing – original draft. HC: Data curation, Investigation, Writing – review & editing. FZ: Writing – review & editing. JL: Conceptualization, Funding acquisition, Supervision, Writing – review & editing.

## Footnotes

^1^ http://www.majorbio.com

^2^ https://www.r-project.org/
