# Supplementary material for: Microbial community structure in an uranium-rich acid mine drainage site: implication for the biogeochemical release of uranium
Source: Front Microbiol. 2024 Jun 26;15:1412599. doi: 10.3389/fmicb.2024.1412599 (PMC11238263; doi:10.3389/fmicb.2024.1412599)
Supplement: Supplementary file 2 [file Data_Sheet_1.PDF]

Supplementary Material

Microbial community structure in an uranium-rich Acid Mine Drainage site: Implication for the biogeochemical release of uranium

Xinxiang Wei, Hongliang Chen, Fangfang Zhu, Jiang Li[*]

*** Correspondence:**

Jiang Li

li66001@aliyun.com

Contents

S1. Supplementary figures……………………………………..……………….……2

S2. Supplementary tables……………………………………………………………..5

S1. Supplementary figures

In this paper, there are 3 supplementary figures.

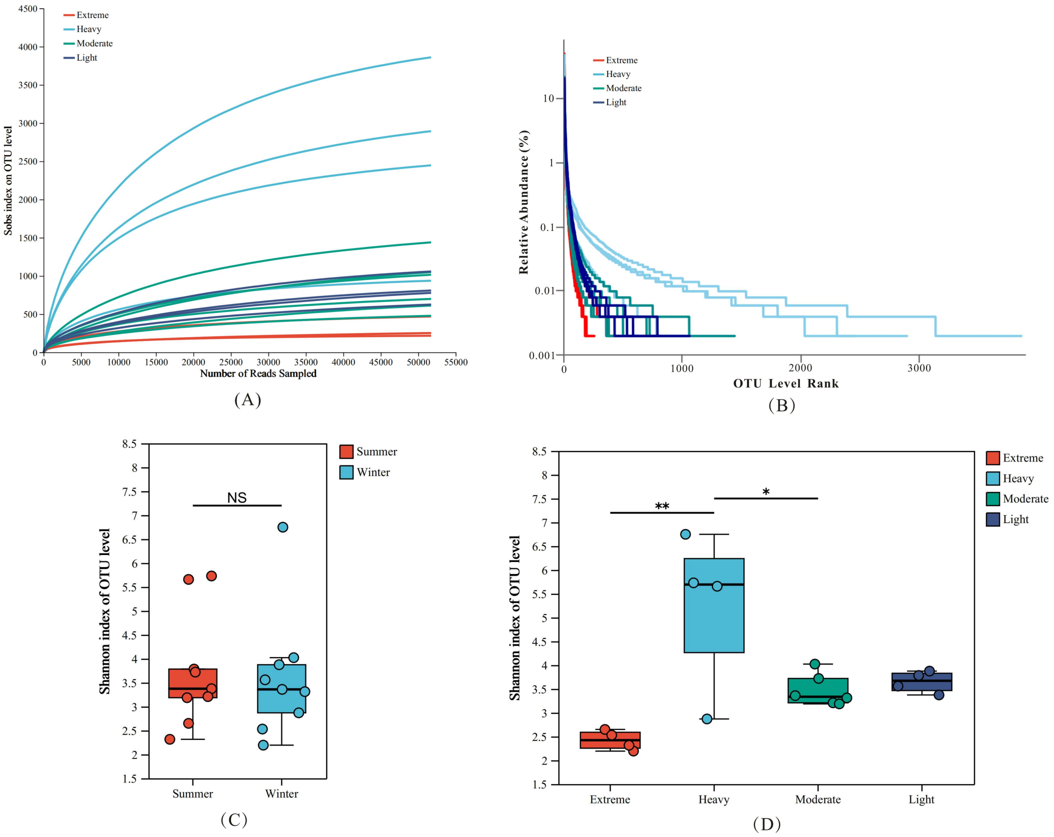

Figure S1 Analysis of bacterial alpha diversity in different groups of samples. (A) Rarefaction curves at different sequencing depths. The horizontal axis represents the amount of randomly sampled sequencing data and the vertical axis represents the Sobs index on OTU level. (B) Relative abundance of the ranking OTU sequences. (C) Student's T test for Shannon index among two sampling seasons. (D) Kruskal-Wallis rank-sum test for Shannon index among four groups. Statistically different groups are showed by * ($P < 0.05$) or ** ($P < 0.01$) or NS (no statistical difference).

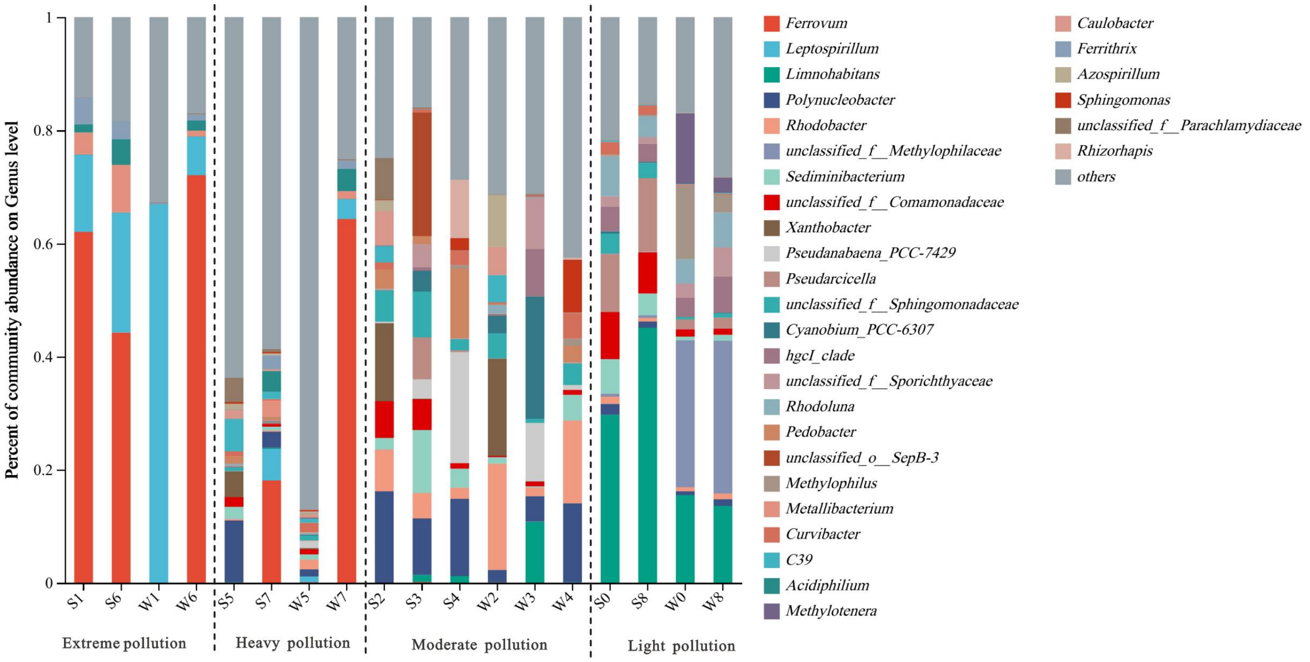

Figure S2 Microbial community structure in water samples at genus level, which showed the abundances of the top 30 genera along the pollution groups.

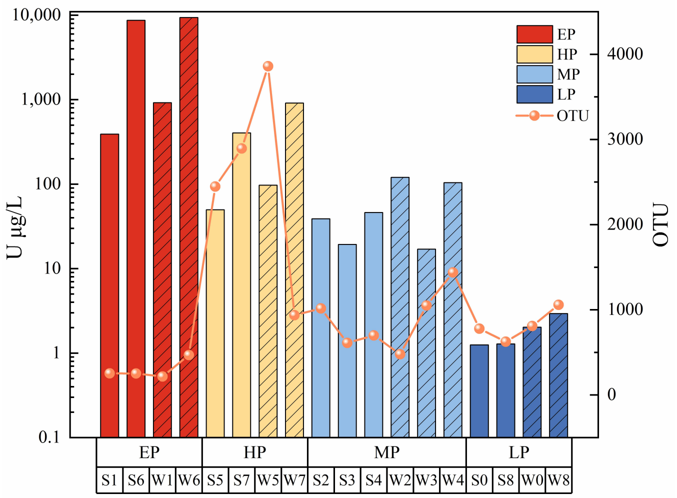

Figure S3 The concentration of uranium (bar chart) and bacterial OTU (line chart) in water samples along the pollution groups. The bars fill with twill refer to samples collected in winter. Abbreviation: EP, extreme pollution; HP, heavy pollution; MP, moderate pollution; LP, light pollution.

S3. Supplementary tables

In this paper, there are a total of 3 supplementary tables and all tables were presented in an Excel file (See Supplementary Tables.xlsx).

Table S1 Statistical results of physicochemical parameters of water samples in four groups. The values are presented as mean ± standard deviation. The values for the two sampling seasons are identified in different colors. Abbreviation: TP, total phosphorus; T, temperature; EC, conductivity; EP, extreme pollution; HP, high pollution; MP, moderate pollution; LP, light pollution. Bdl, below detectable limit.

Table S2 Results of Mantel test analysis. The r represents the statistical value, where the value closer to 1 indicates a positive correlation; closer to -1 indicates a negative correlation; and 0 indicates no correlation. The P-value magnitude is used to measure the level of confidence in the test. Abbreviation: TP, total phosphorus.

Table S3 Relative abundances (%) of uranium-tolerant bacteria in different groups.
